# Ways to Address Perinatal Mast Cell Activation and Focal Brain Inflammation, including Response to SARS-CoV-2, in Autism Spectrum Disorder

**DOI:** 10.3390/jpm11090860

**Published:** 2021-08-29

**Authors:** Theoharis C. Theoharides

**Affiliations:** 1 Laboratory of Molecular Immunopharmacology and Drug Discovery, Department of Immunology, Tufts University School of Medicine, 136 Harrison Avenue, Suite 304, Boston, MA 02111, USA; theoharis.theoharides@tufts.edu; Tel.: +1-(617)-636-6866; Fax: +1-(617)-636-2456; 2 School of Graduate Biomedical Sciences, Tufts University School of Medicine, Boston, MA 02111, USA; 3 Department of Internal Medicine, Tufts University School of Medicine and Tufts Medical Center, Boston, MA 02111, USA; 4 Department of Psychiatry, Tufts University School of Medicine and Tufts Medical Center, Boston, MA 02111, USA

**Keywords:** amygdala, autism spectrum disorder, brain, COVID-19, children, cytokines, flavonoids, inflammation, luteolin, mast cells, microglia, SARS-CoV-2, stress

## Abstract

The prevalence of autism spectrum disorder (ASD) continues to increase, but no distinct pathogenesis or effective treatment are known yet. The presence of many comorbidities further complicates matters, making a personalized approach necessary. An increasing number of reports indicate that inflammation of the brain leads to neurodegenerative changes, especially during perinatal life, “short-circuiting the electrical system” in the amygdala that is essential for our ability to feel emotions, but also regulates fear. Inflammation of the brain can result from the stimulation of mast cells—found in all tissues including the brain—by neuropeptides, stress, toxins, and viruses such as SARS-CoV-2, leading to the activation of microglia. These resident brain defenders then release even more inflammatory molecules and stop “pruning” nerve connections, disrupting neuronal connectivity, lowering the fear threshold, and derailing the expression of emotions, as seen in ASD. Many epidemiological studies have reported a strong association between ASD and atopic dermatitis (eczema), asthma, and food allergies/intolerance, all of which involve activated mast cells. Mast cells can be triggered by allergens, neuropeptides, stress, and toxins, leading to disruption of the blood–brain barrier (BBB) and activation of microglia. Moreover, many epidemiological studies have reported a strong association between stress and atopic dermatitis (eczema) during gestation, which involves activated mast cells. Both mast cells and microglia can also be activated by SARS-CoV-2 in affected mothers during pregnancy. We showed increased expression of the proinflammatory cytokine IL-18 and its receptor, but decreased expression of the anti-inflammatory cytokine IL-38 and its receptor IL-36R, only in the amygdala of deceased children with ASD. We further showed that the natural flavonoid luteolin is a potent inhibitor of the activation of both mast cells and microglia, but also blocks SARS-CoV-2 binding to its receptor angiotensin-converting enzyme 2 (ACE2). A treatment approach should be tailored to each individual patient and should address hyperactivity/stress, allergies, or food intolerance, with the introduction of natural molecules or drugs to inhibit mast cells and microglia, such as liposomal luteolin.

## 1. Introduction

ASD is characterized by difficulties in communication and apparently purposeless repetitive movements [1,2,3,4,5]. The prevalence is estimated to be 1 in 54 children in the United States [6,7] and is associated with enormous economic burden [8,9,10,11]. However, ASD pathogenesis is still unknown. Moreover, most children with ASD have a number of comorbidities such as hyperactivity, gastrointestinal problems, allergies, and seizures [12,13,14], making the development of effective treatments difficult and prompting the need for a personalized approach [15].

A number of risk factors during gestation [16], especially pre-eclampsia [17,18,19], preterm birth, and low birth weight [20,21,22], as well as atopic conditions, autoimmune diseases, [23,24,25] infection, and psychological stress, have been increasingly associated with higher risk of ASD in the offspring (Table 1) [26,27]. There have been many reports of different aspects of immune dysfunction in ASD [28,29,30,31,32]. In fact, maternal antibodies have been implicated in brain pathology in ASD [33], especially autoantibodies against proteins in the developing fetal brain [34,35,36]. We had proposed that focal inflammation in the amygdala may contribute to ASD [37,38,39] via activation of microglia [40,41,42,43]. The present manuscript is organized in different parts, stressing certain risk factors such as SARS-CoV2 infection, psychological stress, atopic conditions, and finally, treatment approaches.

## 2. Infections and COVID-19

Infections [58,60,61] and high fever [58,59] during gestation have been associated with higher risk for ASD. However, there is very little information available on the effect of viruses, especially SARS-CoV-2, on the fetus. Viral proteins can interact with placenta cells [73]. One recent paper that reviewed findings from 101 women infected with SARS-CoV-2 reported that there is vertical transmission of SARS-CoV-2 from the mother to the infant, with adverse effects on the newborn [74]. However, two other papers reported negligible transmission [75,76]. However, transmission may not be required for the virus to induce neuroinflammation, as it may affect peripheral nerves [77] or the developing brain via the Spike protein directly affecting brain cells [78].

Recent publications reported increased perinatal complications in mothers infected with SARS-CoV-2 [56,79], especially pre-eclampsia [79] and premature birth [56,79], associated with inflammatory responses [80,81]. Pre-eclampsia is characterized by high levels of corticotropin-releasing hormone (CRH) [82,83], which is typically secreted from the hypothalamus under stress [84]. With respect to children infected with SARS-CoV-2, even though they have milder pulmonary symptoms than adults [85,86,87,88,89,90,91], a number of papers have reported the presence of Multisystem Inflammatory Syndrome in children (MIS-C) [92,93,94] and adolescents [95]. In such cases, symptoms typically occur 4–6 weeks after infection and are reminiscent of Kawasaki disease [96] but also include neurologic involvement [97]. Moreover, the clinical presentation is associated with elevated markers of inflammation and the presence of multiple autoantibodies [98], and one paper suggested that MIS may be a form of mast cell activation syndrome (MCAS) presenting with neuropsychiatric symptoms and brain fog [57]. In fact, perinatal brain inflammation [99] can contribute to the pathogenesis of neuropsychiatric disorders [100,101], including ASD [16,38,102]. A recent NIH study reported blood vessel damage and perivascular inflammation in brains of deceased patients with COVID-19 [103].

COVID-19 has been associated with neurological [104,105,106,107,108,109,110,111,112], neurodegenerative [107,113], and mental [114,115,116,117,118,119,120,121,122,123,124] disorders, including ASD [125]. Moreover, it is now recognized that as many as 50% of those infected with SARS-CoV-2 [126] develop a post-acute syndrome known as “long-COVID syndrome” [127,128,129]. This syndrome is particularly associated with neurologic and psychiatric symptoms, especially brain fog, [128,130,131,132], as well as persistent fatigue apparently independent of the severity of the initial symptoms [133]. In fact, the Simons Fnd. (New York, NY, USA) recently announced the funding of longitudinal studies of mothers infected with maternal COVID-19 for increased risk for ASD. (https://www.sfari.org/grant/maternal-covid-19-as-a-potential-risk-for-autism-supplemental-funding-for-ongoing-pregnancy-cohorts-request-for-applications/ (accessed on 1 June 2021).

**Figure 1:**
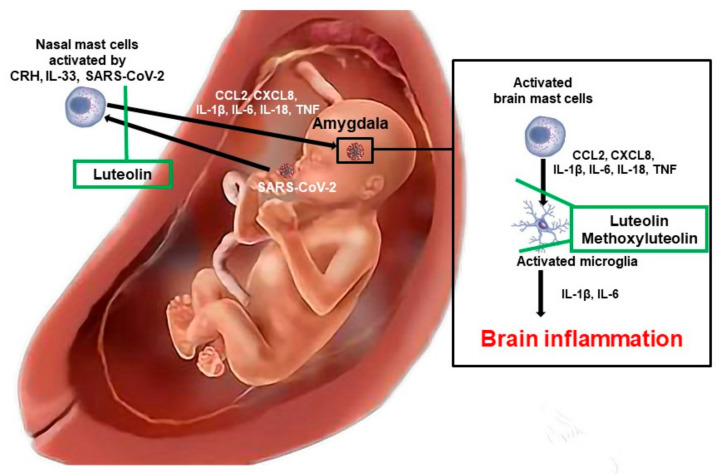
Diagrammatic representation of how SARS-CoV-2 could stimulate fetal mast cells and result in inflammation of the brain. SARS-CoV-2 could stimulate fetal or neonatal mast cells especially in the nose and enter the brain via the olfactory nerve tract, reaching the amygdala. There, it could further activate mast cells and microglia to release pro-inflammatory mediators, thus contributing to brain inflammation and ASD. Luteolin could block these processes.

The detrimental effects of stress, inflammation, and auto-immunity were discussed recently [134], especially with respect to COVID-19 [113] and mast cells [135]. A number of subsequent reviews have discussed neurobiological aspects [136] and neuroinflammation in the context of ASD [137,138,139]. In this paper, we discuss how environmental and stress stimuli trigger fetal or neonatal mast cells to secrete proinflammatory mediators, leading to focal inflammation in the amygdala, regulating emotions and fear (Figure 1) [140] and contributing to ASD [38,45,141]. We further propose a set of laboratory tests and approaches to better identify comorbidities and help each individual to be the best they can be.

## 3. Psychological Stress

Psychological stress can have pro-inflammatory effects [64,134] via CRH [142] stimulating mast cells [135]. One study showed that prenatal and early postnatal stress were associated with elevated serum levels of IL-6 in humans [143]. Another study reported that acute psychological stress increased the circulating levels of proinflammatory cytokines [144]. A longitudinal study of mothers’ serum measurements during gestation linked IL-6 to decreased executive function in their offspring [145]. We had shown that acute restraint stress significantly increased serum IL-6 in mice, which was entirely dependent on mast cells [146]. It is interesting that IL-6 has also been reported to promote human mast cell production and reactivity [147]. Moreover, prenatal stress or exposure to IL-6 resulted in increased microglia ramification in mice and was prevented by IL-6 blockade [148].

Psychological stress could also lead to increased vascular permeability [135]. This process also contributes to the disruption of the blood–brain barrier (BBB) [149,150] via release of CRH [151] and IL-6 [152], permitting entry into the brain of viral particles, cytokines, or other toxic substances, thus further exacerbating brain inflammation. Breakdown of the BBB has been reported in the developing brain following inflammation [153]. We further showed that restraint stress in rodents increased BBB permeability [149,150,154,155] via CRH stimulating mast cells [154,156,157]. The BBB typically prevents circulating toxic substances, but also immune cells, from entering the brain. The BBB is not fully developed until the third trimester [158,159,160] and is more vulnerable to toxins and drugs [161]. It was recently shown that common drugs such as acetaminophen (paracetamol) and cimetidine can enter the fetal brain in higher amounts than the adult brain [162]. Moreover, umbilical cord blood biomarkers indicative of acetaminophen exposure were significantly associated with the risk of ASD in childhood [163]. Hence, many atopic or pathogenic conditions, including exposure to certain drugs, could influence brain development during pregnancy or even lactation.

Stress associated with COVID-19 [134] can further affect the emotional state of individuals [118,119,164,165,166,167], especially social isolation, loneliness, and anxiety [168]. One study reported that prenatal stress was linked to higher risk of newborns developing attention-deficit hyperactivity disorder (ADHD) [65,66] and ASD [67,68,69,70,71,72]. A more recent study of 1638 pregnant women concluded that a high level of perceived stress through pregnancy, especially during the second trimester, was associated with an increased risk of the offspring developing ASD at 6 months of age [62]. Prenatal stress may lead to maternal immune dysregulation, thus contributing to ASD [70]. It is interesting that maternal psychological stress during pregnancy increased cord blood levels of IgE [169], suggesting that it could contribute to an increased risk in the fetus of developing allergic reactions or sensitivity to postnatal exposure to allergens. Psychological stress also increased the risk of childhood atopic dermatitis (AD) [170,171] and asthma [172,173,174]. To make matters worse, children with ASD cannot handle stress [175,176] and have an exacerbated sense of fear [39].

## 4. Mast Cell Activation

Infection with SARS-CoV-2 is primarily characterized by the release of a storm of pro-inflammatory cytokines [177,178,179,180,181,182,183,184,185], especially IL-6 [186,187,188,189] and IL-1β [190,191]. Mast cells are a key source of such cytokines in COVID-19 [192,193,194,195] and could contribute to interstitial lung edema and immunothromboses [196].

We reported that children born to mothers with systemic mastocytosis [63], which is characterized by a greater number of hyperactive mast cells than in the general population [197], had a higher risk of developing ASD [1,2,7,198,199]. The word atopy is commonly used to denote a tendency, usually early in life, to become sensitized to and produce immune IgE to environmental antigens. Many epidemiological studies reported a strong association between atopic diseases and behavioral problems in general [200] and in ASD in particular [46,47]. Other epidemiological studies showed a strong association between risk for developing ASD and allergies [45,46,48,49,50], especially asthma [50,53] and atopic dermatitis (AD) [54], but also food hypersensitivity [12,201,202,203,204,205]. In fact, the presence of allergies was associated with elevated serum levels of autoantibodies against brain antigens in children with ASD [206]. Parental history of AD was strongly associated with children developing AD [207]. It was reported that maternal immune activation [208] and autoimmune diseases [209], especially psoriasis, but also allergies and asthma, were associated with a higher risk of ASD [23]. In another study, almost 50% of children with ASD had relatives with rheumatoid diseases as compared to 26% in the control group [210]. In a recent large study, mothers who suffered from asthma, allergy, atopy, or eczema during pregnancy were associated with a higher risk of neuropsychiatric problems in children [55]. Three recent studies reported strong associations with ASD and food allergy [211] and food intolerance [202] that could lead to brain inflammation and cognitive impairment [212].

A recent publication showed that the mother’s circulating immune IgE resulted in vertical transmission of AD in the newborn via stimulation of fetal mast cells [213]; both passive and active prenatal sensitization conferred allergen sensitivity [213]. This important paper indicated that fetal mast cells were functional and could be stimulated by specific IgE and allergens present in the mother during gestation. Even though these studies were limited to pulmonary and skin mast cells, reactivity could also extend to brain mast cells. In fact, prenatal allergen exposure was even shown to program lifelong changes in adults rats’ social and sexual behavior, including effects on microglia activation and neonatal dendritic spine density [214]. Fetal mast cells could potentially respond to other stimuli such as neuropeptides and toxins, including the alarmin IL-33 [215,216], with detrimental effects on brain development, especially in premature babies [16].

Activated brain mast cells have been shown to contribute to cognitive dysfunction via microglia activation and neuronal apoptosis [217]. Mast cells are ubiquitous in the body [218] and are critical for allergic diseases [219], including mastocytosis [197]. However, mast cells also participate in inflammation [220,221] by secreting histamine and multiple pro-inflammatory cytokines and chemokines [222,223], including IL-1β [224], IL-6 [225], and TNF [226]. Mast cells are also present in the brain, especially the meninges [227,228] and the median eminence [229], where they are located perivascularly, close to nerve endings positive for CRH [227]. We showed that stress stimulates mast cells via CRH [135] leading to increased dura vascular permeability, an effect that was absent in mast cell-deficient mice [230]. Moreover, mast cells can activate the hypothalamic–pituitary–adrenal (HPA) axis [142,231,232,233] via the release of histamine [234], IL-6 [152], and CRH [151]. Moreover, neurotensin [235] and substance P (SP) [236], neuropeptides implicated in inflammation, induced CRHR-1, thus creating an autocrine loop. Moreover, SP induced the ST2 receptor for IL-33 [226], further exacerbating mast cell activation by the combined action of neuropeptides and IL-33.

Mast cells respond not only to allergic but also to many other stimuli that can act alone or increase mast cell reactivity [197]. Mast cells can also be triggered by viruses [237] including SARS-CoV-2 [192,195]. In fact, gene expression of the coronavirus surface receptor angiotensin-converting enzyme 2 (ACE2) was recently shown to be induced by interferon [238], and mast cells can elicit strong pro-inflammatory and Type I interferon responses in the presence of viruses [239], implying an autocrine action on ACE2 expression. Following stimulation, mast cells release large amounts of pro-inflammatory mediators [222] such as histamine, tryptase, chemokines (e.g., CCL2, CCXL8) [240], and cytokines (IL-6, [225] IL-1β [224], TNF [226]), especially when primed by IL-33 [216,241]. Histamine can stimulate macrophages to release IL-1 [242], which in turn stimulates mast cells to release IL-6 [225]. Mast cells can also secrete mitochondrial DNA (mtDNA) extracellularly [243], which serves as an alarmin and can stimulate pro-inflammatory mediator secretion from immune cells [244,245]. We reported elevated extracellular mtDNA in the serum of children with ASD [246]. In fact, it was recently reported that mtDNA may mediate prenatal environmental influences in ASD [247], was increased in the serum of COVID-19 patients, and correlated with disease severity [248]. Moreover, mast cells synthesize and release platelet-activating factor (PAF), which has been implicated in inflammation [249] and microthromboses [250] characterizing COVID-19. In fact, a recent paper reported a strong association across the globe with SARS-CoV-2 infection rates and levels of pollen known to be involved in upper respiratory system allergies, thus implicating mast cell activation [251]

## 5. Mast Cells and Microglia

Microglia are specialized resident macrophages of the Central Nervous System (CNS) with important functions in both health and disease. They are especially implicated in neuroinflammation [252,253,254] and neurodegenerative [252,255,256,257] diseases. Activation of microglia has been reported in ASD [41,42,43,258], as documented by the release of the pro-inflammatory mediators IL-1β and CXCL8 [259]. Microglia were recently implicated in COVID-19 [260] and were also associated with neuroinflammation [261]. The transition of microglia from the resting to the activated proinflammatory phase is regulated by several intrinsic and extrinsic factors. Microglia can be activated by numerous molecules including pathogen-associated molecular patterns (PAMPs) and endogenous damage-associated patterns (DAMPs) acting on Toll-like receptors (TLRs), but also in response to molecules released from mast cells, such as histamine and tryptase (Table 2) [39]. It was recently reported that elevated protein synthesis in microglia resulted in autism-like synaptic and behavioral changes in mice [262]. A dysfunctional neuroimmune cross-talk may result in a state of chronic fetal microglial activation leading to a disruption of neurogenesis and synaptic pruning [263], processes critical for the development of ASD.

Mast cells interact with microglia in the brain [264], leading to their activation [264,265,266,267] and to neuroinflammation [266,268]. This effect is absent in mast cell-deficient mice [39,269]. Activation of mast cells [270,271] and microglia [272], especially in the hypothalamus [273], could lead to cognitive dysfunction [274]. Microglia express receptors for CRH [275] and could be further activated by stress, especially in association with COVID-19 [276]. Microglia also express receptors for neurotensin (NT) (Table 2) [277]. We reported that NT is increased in the serum of patients with ASD [278,279] and can activate human microglia to secrete pro-inflammatory molecules [259]. We also reported increased gene expression of the pro-inflammatory microRNA-155 (miR-155) in the amygdala of children with ASD [280], as well as reduced expression of the anti-inflammatory cytokine IL-38 [281]. Microglia also express TLRs [282] and were recently implicated in COVID-19 [260,283].

## 6. Treatment Approaches

It is critical to identify the presence of any atopy or allergies and food intolerance, especially the presence of Mast Cell Activation Syndrome (MCAS) [284,285] or systemic mastocytosis (SM) [197], by measuring the levels of the molecules listed in Table 3. Of note is IgG4 because it is involved in food intolerance and has been reported to be elevated in the plasma of children with ASD [286].

It is also important to avoid histamine-rich foods, especially ripe tomatoes and avocados, cheeses, spinach, tangerines, spices, and sardines, which have been associated with histamine intolerance [287]. In this context, it is useful to conduct gene analysis for metabolizing enzymes, especially diamine oxidase (DAO), which breaks down histamine, and enzymes that break down phenols such as monoamine oxidase (MAO), catecholamine-ortho-methyl transferase (COMT), and phenol sulfur transferase (PST) to ascertain phenol intolerance that can contribute to hyperactivity. If DAO gene expression is defective and/or its activity in the blood is low, DAO supplements can be added about 30 min before meals, but one should be careful to avoid the common dyes and preservatives mentioned below.

Unfortunately, many medications, supplements, and vitamins contain “inactive” ingredients that are not tolerated by many children with ASD, leading to unexpected or worsening of behaviors. Such ingredients to be avoided include dyes, preservatives, gluten, monosodium glutamate (MSG), polyethylene glycol (PGE), galactosaccharide (GOS), salicylates, silicum, soy talc, and Twin 80. In addition, herbicides such as glyphosate and atrazine should be avoided, as they have been reported to stimulate mast cells and promote inflammation [288], besides their known neurotoxic effects.

One should choose the best tolerated antihistamine [289,290] from the list shown in Table 4, especially rupatadine, which also blocks mast cells, [291,292,293], and avoid large doses that may lead to confusion [294]. In fact, the Food and Drug Administration (FDA) recently warned that taking higher-than-recommended doses of diphenhydramine (Benadryl) can lead to serious heart problems, seizures, coma, or even death. https://www.fda.gov/drugs/drug-safety-and-availability/fda-warns-about-serious-problems-high-doses-allergy-medicine-diphenhydramine-benadryl (accessed on 1 June 2021).

As discussed, anxiety, fear, and stress are major factors leading to hyperactivity. This should be investigated (by measuring total blood catecholamines and glutamate) and addressed with the use of a chamomile/passiflora/valerian extract or Ashwagandha [295,296]. If these are not sufficient, one should consider the beta-blocker propranolol that has good anti-anxiety properties without clouding the mental abilities and has also been reported to improve language in children with ASD [297]. Alternatively, one may recommend the use of alpha 2-receptor agonists [298] such as clonidine [299,300] and guanfacine [301,302], usually administered at bedtime especially since clonidine reduces sleep initiation latency and night awakening [303]. Moreover, caution should be exercised because such adrenergic blocking drugs may cause bradycardia and a drop in blood pressure. Cannabidiol (CBD) oil may be useful but it should be used with caution in individuals with atopic problems, because it has been reported to trigger the activation of cultured leukemic mast cells [304].

There has been considerable progress in defining drugs that block tyrosine kinases (TK) that are involved in mast cell proliferation [305]. The use of biologics for TNF [306,307] and IL-1β, [308]; has significantly improved the treatment of inflammatory skin diseases. However, these agents have a number of limitations as they may cause paradoxical inflammation, reduced ability to fight infection, and cancer development [309]. In spite of such advances, there is no clinically effective inhibitor of human mast cell mediator secretion. Moreover, inhibitors of the tyrosine kinase c-kit receptor that reduce MC proliferation [310] do not inhibit mast cell activation [311]. There are still no clinically effective mast cell inhibitors [221,312]. Disodium cromoglycate (cromolyn), known as a “mast cell stabilizer,” had originally been shown to inhibit rat peritoneal MC histamine release [313]. However, cromolyn does not effectively inhibit either murine MC [314] or human MC [315,316,317] and has even been reported to potentiate histamine release from mast cells [318].

Instead of cromolyn, one should choose the best purity, source, and formulation of the flavonoids luteolin and quercetin [319,320,321,322,323]. These flavonoids are readily available and are generally considered safe [45,324,325,326]. Luteolin has broad anti-viral properties [327,328,329] and inhibits the entry of the corona virus into host cells [237,330,331]. Furthermore, luteolin better penetrates into the brain, inhibits both microglia [259,332,333,334] and mast cells [317,335], is neuroprotective [336,337,338,339], and has been reported to reduce neuroinflammation [337,340,341,342] and cognitive dysfunction [61,343,344,345], especially brain fog [346]. In fact, flavonoids were recently shown to improve cerebral cortical oxygenation and cognition in healthy adults. [347,348] Moreover, flavonoids induce the synthesis and secretion of neurotrophic factors, including brain-derived neurotrophic factor (BDNF) [77,349,350], known to be deficient in certain conditions associated with ASD, such as RETT syndrome [351]. The beneficial actions of luteolin are summarized in Table 5.

Luteolin and quercetin are not water-soluble and are difficult to absorb in powder form after oral administration [357], but their intestinal uptake can be greatly improved [358] in liposomal preparations using olive pomace oil [358]. In fact, such a luteolin formulation in olive pomace oil (NeuroProtek^®^) has been reported to improve ASD [359,360], while another one (BrainGain^®^) reduced brain fog [344]. The latter formulation also provided the additional neuroprotective [361,362,363,364,365,366] and anti-inflammatory [367,368] actions of olive pomace oil polyphenols, as well as the increase in memory induced by the olive oil component hydroxytyrosol [365,369].

The beneficial actions of these supplements could be combined with that of a unique, hypoallergenic skin lotion containing tetramethoxyflavone (GentleDerm^®^) [305], which can be applied on the forehead for direct absorption by temporal blood vessels. Tetramethoxyflavone (methoxyluteolin, methlut) is a more potent inhibitor of human mast cells than either quercetin of luteolin [317,335] and also inhibits human microglia [259,333].

The natural molecule berberine may be particularly useful in cases of PANS/PANDAS because of its antibacterial properties, but also because it can inhibit mast cells [370,371] and improve brain circulation [372]. In addition, high doses of Vitamin D3 are recommended, because this vitamin has been found to be present at low levels in mothers and/or children with ASD [371,373,374,375] and also decreases atopic responses [376]. When all fails, intravenous Ig may be administered [289].

## 7. Conclusions

It is critical to try to address each child individually (Table 6) [377] by first identifying any comorbidity, especially atopic diseases and hyperactivity, as well as any metabolic issue especially related to vitamins B1, B6, B12, folic acid/MTHFR, thyroid, or vitamin D3 deficiency, since these may be easily overcome. Inflammation of the brain may be reduced with the use of the natural flavonoid luteolin, especially when formulated in liposomal form in olive pomace oil that significantly increases oral absorption (BrainGain^®^, PureLut^®^, NeuroProtek^®^ with FDA Certificate of Free Sale). The beneficial actions of these supplements could be augmented by the use of a unique, hypoallergenic skin lotion (GentleDerm^®^), which contains the more potent methoxyluteolin and can be applied on the temples for direct absorption by brain blood vessels. Thus, inhibiting the activation of mast cells and microglia not only would prevent vertical transmission of atopic disorders, but also may prevent inflammation of the brain and reduce the risk of the offspring developing neuropsychiatric disorders, especially ASD (US patents US 7,906,153; 8,268,365; 9050275).

## Figures and Tables

**Table 1:** Conditions Associated with Higher Risk of ASD.

• Autoimmunity	[24,25,44]
• Allergies	[45,46,47,48,49,50,51,52]
• Asthma	[50,53]
• Atopic dermatitis	[54,55]
• COVID-19	[56,57]
• High fever	[58,59]
• Hypothyroidism	[24]
• Infection	[58,60,61]
• Inflammation	[38,39]
• Low birth weight/preterm birth	[16,20,21,22,62]
• Pre-eclampsia	[17,18,19]
• Mastocytosis	[63]
• Psoriasis	[23,24,25]
• Rheumatoid arthritis	[24]
• Stress	[16,62,64,65,66,67,68,69,70,71,72]

**Table 2:** Molecules Activating Microglia.

• Chemokines (CCL2, CxCl8)
• Cytokines (IL-1β, IL-6, IL-18, TNF)
• Histamine
• Lipopolysaccharide (LPS)
• Neuropeptides (CRH, HK-1, Neurotensin, SP)
• Neurotransmitters
• Pathogens (SARS-CoV-2)
• Potassium
• Prostaglandin D2
• Proteases (MMP-3, Thrombin, Tryptase)

**Table 3:** Laboratory Tests for Diagnosis of Atopic Diseases.

Blood
• IgA, IgG_1_, IgG_4_, IgE
• Immune IgE (RAST for alpha-gal, casein, dust, dust mites, egg, fungi, grass, gluten, pollen)
• Anti-IgE receptor antibody (basophil activation or histamine release test)
• CCL2, CXCL8 (IL-8)
• Chromogranin A *
• Eosinophilic cationic protein (ECP)
• Food Intolerance Panel
• Heparin
• IL-4, IL-6, IL-31, IL-33
• Prostaglandin D_2_ (PGD_2_)
• Tryptase
Urine collected for 24 h or first morning void (must be kept and sent cold)
• N-methylhistamine (NMH) or methylimidazole acetic acid (MIA)
• PGD_2_
• 23BPG=2,3-Dinor-11β-PGF_2α_

* Should be measured after one week of NO antacids, otherwise there is a high chance of false positive results. Elevated chromogranin A is not indicative of atopy, but of a somewhat similar condition called carcinoid syndrome associated with activated enterochromaffin cells in the gut.

**Table 4:** Different histamine-1 receptor antagonists.

Generic Drug	(Trade Name)
Bilastine *	Nonsedating, non-metabolized
Cetirizine	Nonsedating
Cyproheptadine	Antiserotonergic
Diphenhydramine	Sedating
Hydroxyzine	Anxiolytic
Ketotifen *	Anti-eosinophilic
Loratadine	Nonsedating
Rupatadine *	Anti-PAF (Platelet activating factor), mast cell inhibitor
Tricyclic Antidepressants	
Amitriptyline	Weight gain
Doxepin	Also histamine-2 receptor antagonist
Phenothiazines	
Promethazine	Antiemetic
Prochlorperazine	Antiemetic, mast cell inhibitor

* Available only via compounding in the United States.

**Table 5:** Beneficial Actions of Luteolin *.

• Antagonizes SARS-CoV-2 Spike protein binding	[237,330,331,352]
• Has broad antiviral properties	[327,328,329]
• Improves cerebral cortical oxygenation and cognition	[347,348]
• Induces the synthesis and secretion of BDNF	[77,349,350,351]
• Inhibits serine proteases required for Spike protein processing	[353,354]
• Inhibits neuroinflammation	[340,341,342]
• Inhibits the release and action of PAF	[249,250]
• Inhibits mast cell stimulation by different triggers	[317,335]
• Inhibits microglia activation	[259,332,333,334]
• Interferes with coronavirus replication	[355]
• Is neuroprotective	[336,337,338,339]
• Reduces cognitive decline	[61,343,344,345,346]
• Reduces oxidative stress	[320]
• Regulates inflammasome activation	[356]

* Methoxyluteolin is more potent, metabolically stable, enters the brain more efficiently, and is better tolerated due to the absence of phenolic groups.

**Table 6:** Treatment Approaches.

Hyperactivity
• Ashwagandha
• Chamomile/Passiflora/Valerian extract
• Clonidine or guanfacine
• C-Acetyl cysteine (NAC)
• Hydroxyzine
• Propranolol
Allergic Inflammation
• Berberine
• Luteolin *
• Rupatadine
• Vitamin D3
Neuronal fatigue
• Folinate calcium or methylfolate
• Glutathione
• Methyl B12
• S-Adenosylmethionine (SAMe)
OCD
• Aripiprazole
• Risperidone

* Children with phenol intolerance: PureLut^®^; NeuroProtek-Low Phenol^®^, Adults with Brain Fog: BrainGain^®^; NeuroProtek^®^, Adults also with allergies: FibroProtek^®^, Adults also with interstitial cystitis: CystoProtek^®^.

## Data Availability

Not applicable.

## Abbreviations

ACE2	Angiotensin-converting enzyme 2
ADHD	Attention Deficit Hyperactivity Disorder
BBB	Blood–brain barrier
BDNF	Brain-derived neurotrophic factor
CNS	Central nervous system
CRH	Corticotropin-releasing hormone
DAMPs	Damage-associated molecular patterns
HPA	Hypothalamic–pituitary–adrenal
MCAS	Mast Cell Activation Syndrome
MCI	Mild cognitive impairment
mtDNA	Mitochondrial DNA
MIS	Multisystem Inflammatory Syndrome
NT	Neurotensin
PANS	Pediatric Acute Neuropsychiatric Syndrome
PAMPs	Pathogen-associated molecular patterns
PAF	Platelet-activating factor
SP	Substance P
TLR	Toll-like receptor
